# A Novel Energy-from-Waste Approach for Electrical Energy Production by Galvano–Fenton Process

**DOI:** 10.3390/molecules26134013

**Published:** 2021-06-30

**Authors:** Intissar Gasmi, Naoufel Haddour, Oualid Hamdaoui, Kaouther Kerboua, Abdulaziz Alghyamah, François Buret

**Affiliations:** 1 Laboratoire Ampère, École Centrale de Lyon, 36 Avenue Guy de Collongue, 69134 Ecully, France; gasmiintissar7@gmail.com (I.G.); francois.buret@ec-lyon.fr (F.B.); 2 Laboratory of Environmental Engineering, Process Engineering Department, Faculty of Engineering, Badji Mokhtar-Annaba University, P.O. Box 12, Annaba 23000, Algeria; ohamdaoui@yahoo.fr (O.H.); k.kerboua@esti-annaba.dz (K.K.); 3 Chemical Engineering Department, College of Engineering, King Saud University, P.O. Box 800, Riyadh 11421, Saudi Arabia; aalghyamah@ksu.edu.sa; 4 Department of Second Cycle, Higher School of Industrial Technologies, P.O. Box 218, Annaba 23000, Algeria

**Keywords:** Galvano–Fenton process, new energy sources, micropollutants, ferrous scrap recovery, electrochemical wastewater treatment

## Abstract

A novel approach allowing the production of electrical energy by an advanced oxidation process is proposed to eliminate organic micropollutants (MPs) in wastewaters. This approach is based on associating the Galvano–Fenton process to the generation of electrical power. In the previous studies describing the Galvano–Fenton (GF) process, iron was directly coupled to a metal of more positive potential to ensure degradation of organic pollutants without any possibility of producing electrical energy. In this new approach, the Galvano–Fenton process is constructed as an electrochemical cell with an external circuit allowing recovering electrons exchanged during the process. In this study, Malachite Green (MG) dye was used as a model of organic pollutant. Simultaneous MG degradation and electrical energy production with the GF method were investigated in batch process. The investigation of various design parameters emphasis that utilization of copper as a low-cost cathode material in the galvanic couple, provides the best treatment and electrical production performances. Moreover, these performances are improved by increasing the surface area of the cathode. The present work reveals that the GF process has a potential to provide an electrical power density of about 200 W m^−2^. These interesting performances indicate that this novel Energy-from-Waste strategy of the GF process could serve as an ecological solution for wastewater treatment.

## 1. Introduction

The worldwide contamination of surface water, groundwater, and drinking water with organic micropollutants (MPs) such as cosmetics, pesticides, pharmaceuticals, steroid hormones …, poses potential risks for human health and the ecosystem [1]. Since MPs are resistant to biological degradation, most wastewater treatment plants cannot effectively eliminate them from the treated effluents and cannot prevent them from entering the environment [2]. Therefore, efficient and inexpensive wastewater treatment processes for eliminating MPs are needed. Different methods have been proposed to remove MPs from aqueous solution reviewed in several papers [3,4]. Among these mentioned methods, the destruction of MPs by Fenton reagent is a promising option due to its efficiency and operational simplicity [5]. In Fenton treatment, hydroxyl radical (^•^OH), a strong oxidant of organic pollutants, is produced in the aqueous solution with a mixture of ferrous ion and hydrogen peroxide as expressed in Equation (1).

Fe^2+^ + H_2_O_2_ → Fe^3+^ + ^•^OH + OH^−^ (1)

Other variants of the Fenton process, such as electro-Fenton [6], photo-Fenton [7], and photo-electro-Fenton [8], have a great efficiency in destroying organic pollutants. Indeed, the different forms of energy input enhance the rate of Fenton’s reaction and therefore the degradation of organic pollutants. However, the major drawbacks of these processes are their need for continuous energy consumption, which increases equipment and operating costs. These challenges have been addressed in several recent studies [9,10]. On the other hand, the classical Fenton process requires no additional energy. It is a relatively economical method characterized by its lower equipment costs. However, it requires continuous injection of excessive amounts of ferrous ions during treatment [11]. Moreover, direct use of ferrous salt catalyst results in rapid and useless consumption of Fe^2+^ as well as overload of ferric ions in the treatment medium [12,13]. Therefore, several studies have reported the use of zero valent iron (ZVI) as the heterogeneous source of Fe^2+^ ions in the Fenton process [14,15]. In this technology, ZVI metal in contact with aqueous solution corrodes spontaneously producing ferrous ions [16]. The oxidation of ZVI metal in acidic condition, required in Fenton’s reaction, is usually linked to the reduction of H^+^ producing H_2_ [17]. The global mechanism associated to ZVI-based technology may be represented by Equations (1) and (2).

Fe^0^ + 2H^+^ → Fe^2+^ + H_2_ (2)

**Figure 1:**
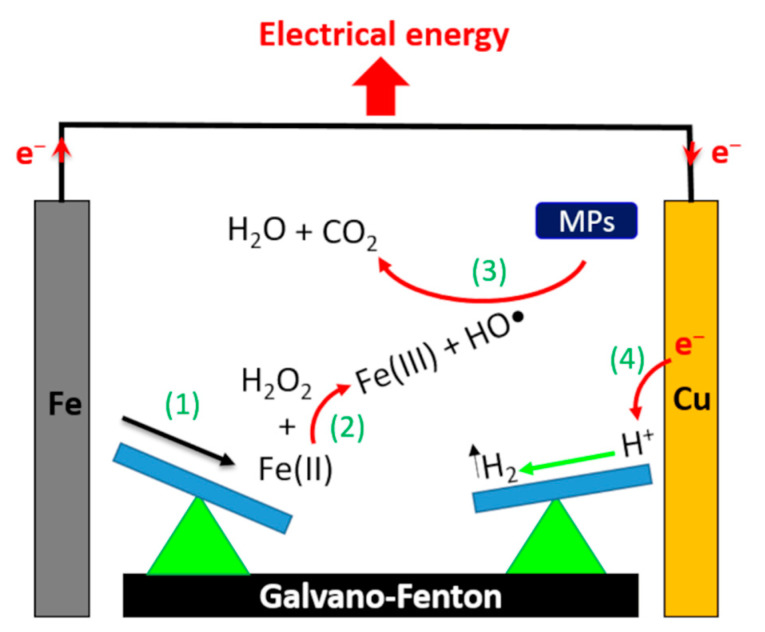
Schematic representation of the mechanism for producing electrical energy with the Galvano–Fenton process.

This approach is a promising alternative since it allows both to overcome the disadvantages associated with Fe^2+^-based Fenton processes and to reutilize scrap iron residues or byproduct from other industries. Indeed, recent studies have reported the use of scrap zero-valent iron (SZVI) as heterogeneous catalysts in the Fenton process to eliminate successfully organic pollutants [18,19]. Therefore, the use of ZVI offers the possibility of reducing treatment costs while recovering waste material. We have recently patented an advanced Fenton process (called Galvano–Fenton (GF)), which assist the Fenton reaction by a galvanic corrosion of iron plates [20]. This new process ensures effective degradation of organic pollutants by continuous in situ production of catalyst without any energy consumption [21,22]. Herein, we describe a new approach to convert the Galvano–Fenton process to a source of electrical power, which increases the efficiency of the Fenton reaction and reduces running costs. In the previous studies describing Galvano–Fenton process, iron was directly coupled to a metal of more positive potential to accelerate iron corrosion without any possibility of producing electrical energy. In this new approach, the Galvano–Fenton process is constructed as an electrochemical cell with an external circuit allowing recovering electrons exchanged during the process (Figure 1). Iron is contacted with a more noble metal that acts as a cathode and the more negative iron metal becomes an anode. In this galvanic cell, iron anode corrodes at an accelerated rate and the cathode is protected from corrosion [23]. Therefore, iron metal is used as a sacrificial anode and is continuously consumed by anodic dissolution reaction, which permits to increase the quantity of ferrous ions produced in solution. Moreover, the galvanic coupling allows electrical energy production. Indeed, due to the driving force resulting from the potential difference between the anode and cathode, electrons produced by iron oxidation circulate spontaneously in the external circuit to the cathode. At this positive electrode, electrons allow reduction of protons forming hydrogen gas. In the meantime, the flow of electrons between both electrodes in an external resistance generates an electrical current that can be harnessed. In the present study, malachite green (MG), a triphenylmethane dye, was used as an organic pollutant model to investigate the effect of different operating parameters on the performance of the GF process. MG has been extensively used as dye in textile industries causing several health hazards [24]. Therefore, many treatment technologies have been applied to study the degradation of MG in aqueous medium [25,26,27].

## 2. Results and Discussion

### 2.1. Effect of Cathode Materials on GF Process Performances

**Figure 2:**
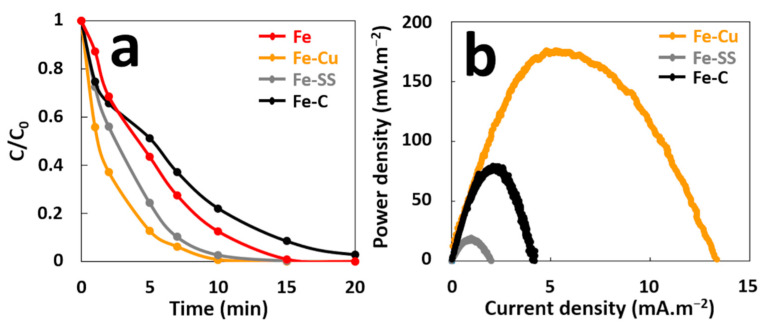
(**a**) Effect of cathode material on the decolorization of MG dye during the GF process, (**b**) Power curves of the GF process obtained with different cathode materials.

Some variables such as corrosion potentials of anode and cathode, as well as their relative surface areas, can affect the rate of the galvanic corrosion [23] and therefore the performance of GF process in terms of pollutant degradation and electrical energy production. First, the effect of cathode material on performance of GF process was examined. In this study, graphite (C), stainless steel (SS), and copper (Cu) electrodes were used as cathodes, since these materials are more noble than iron and easily available at reasonable prices. The degradation of MG (10 mg·L^−1^, 21.57 µM) in aqueous solutions containing 2 mM H_2_O_2_ acidified with sulfuric acid at an initial pH of 3.00, was conducted in a batch reactor in the presence of an iron plate (6 cm^2^ of immersed surface) electrically connected to a cathode of the same surface (Figure S1). Figure 2a shows that galvanic coupling increases initial degradation rate of MG for the GF process obtained with the different cathode materials (Fe–Cu, Fe–SS, and Fe–C) compared to the ZVI-based process without galvanic coupling (Fe). Data inspection reveals that MG degradation occurs more rapidly at the beginning of treatment process and thus, 5.8, 10.6, 11.3, and 19.3 µM·min^−1^ initial degradation rates are obtained for the Fe, Fe–C, Fe–SS, and Fe–Cu processes, respectively (Table S1). Although oxidation capacity is maximum (1/b~1) for all processes, 100% decolorization of MG is attained with different durations of 10, 12, 15, and 20 min for the Fe–Cu, Fe–SS, Fe, and Fe–C processes, respectively. These results indicate that copper material is the best cathode for degradation performances in the GF process.

**Figure 3:**
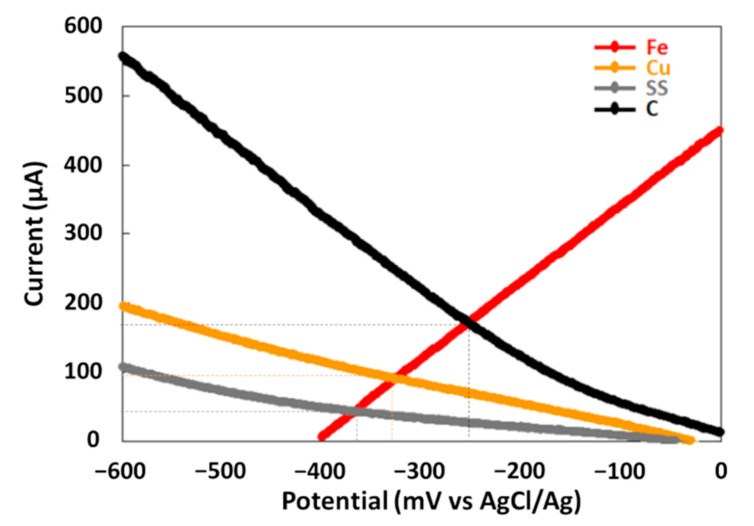
Corrosion potential and current corrosion of iron electrode coupled to different cathodes.

The maximal power density (P_max_) was determined from power curves of each galvanic couple during the treatment in order to compare their electrical energy performances (Figure 2b). P_max_ are different for the three couples. The GF process with the Fe–SS couple produces the lowest P_max_ of 18 mW·m^−2^. The Fe–C couple provides a P_max_ of 80 mW·m^−2^ while the Fe–Cu couple produces the best P_max_ of 175 mW·m^−2^. An Evans diagram was plotted for each galvanic couple (Figure 3). This potential–current density diagram shows that the best cathode material to be connected to iron is graphite (C) since it provides the higher corrosion current (I_corr_ = 27 µA·cm^−2^) with more positive galvanic corrosion potential (E_corr_ = −246 mV/AgCl/Ag). Moreover, the Fe–Cu and Fe–SS galvanic couples provide lower I_corr_ values (Fe–Cu: I_corr_ = 15 µA·cm^−2^ and Fe–SS: I_corr_ = 7 µA·cm^−2^) with more negative E_corr_ values (Fe–Cu: E_corr_ = −325 mV/AgCl/Ag and Fe–SS: E_corr_ = −360 mV/AgCl/Ag) indicating that iron corrodes at a lower rate when it is connected to them. These results disagree with those resulting from the study of MG degradation and electrical energy production with the Fe–C couple. This is probably due to the reaction of hydroxyl radicals during the treatment with the carbonaceous cathode to form simple olefinic and acetylenic hydrocarbons, as previously reported [28]. This reaction leads to the consumption of free radicals by the carbonaceous cathode during the treatment and the loss in its electrochemical activity.

### 2.2. Comparison of Two Different GF Process Configurations

Degradation performances of GF process were examined using 10 mg·L^−1^ of MG solutions under two different conditions (GF-A and GF-B) that can be used for energy production before degradation process (GF-A) or during degradation process (GF-B). In the first condition (GF-A), the iron anode and copper cathode were removed from solutions after different immersion times and H_2_O_2_ was added at a final concentration of 2 mM to initiate the degradation reaction. In the second condition (GF-B), the electrodes were left immersed in solutions during the degradation reaction started by adding H_2_O_2_ after immersion times (Figure S2). To carry out this study, the concentration of total dissolved iron ions produced by galvanic corrosion of an iron plate (6 cm^2^ of immersed surface) electrically connected to a copper cathode of the same surface in aqueous solutions (100 mL) at an initial pH of 3.00, was determined for different immersion times. Thus, 2.7, 8, 11.8, and 17.8 mg·L^−1^ of total dissolved iron concentrations are obtained for 1, 5, 10, and 20 immersion times, respectively. Degradation performances of both GF process conditions were compared to MG degradation obtained by the classical Fenton process (CF) in the same operating conditions for Fe^2+^ concentrations equivalent to the total dissolved iron ions determined for the four immersion times.

**Figure 4:**
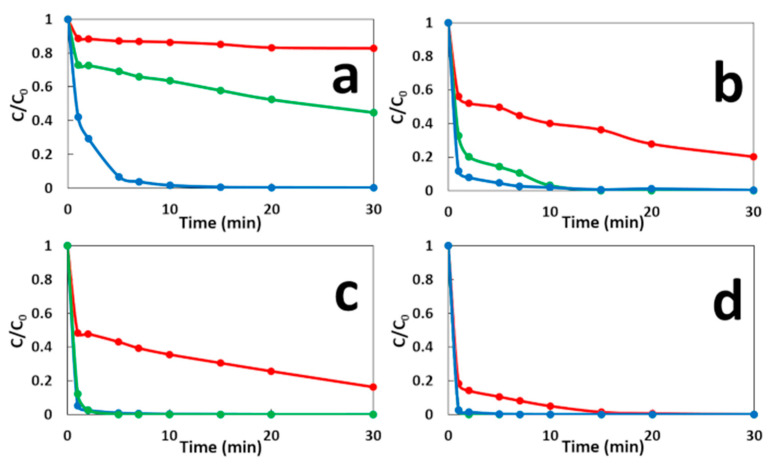
Effect of the cathode material on the decolorization of MG dye during the GF-A process (green line) and the GF-B process (blue line) for: (**a**) 2.7 mg·L^−1^ of Fe^2+^/1 min immersion, (**b**) 8 mg·L^−1^ of Fe^2+^/5 min immersion, (**c**) 11.8 mg·L^−1^ of Fe^2+^/10 min immersion, and (**d**) 17.8 mg·L^−1^ of Fe^2+^/20 min immersion. The CF process (red line) is presented for comparison.

**Figure 5:**
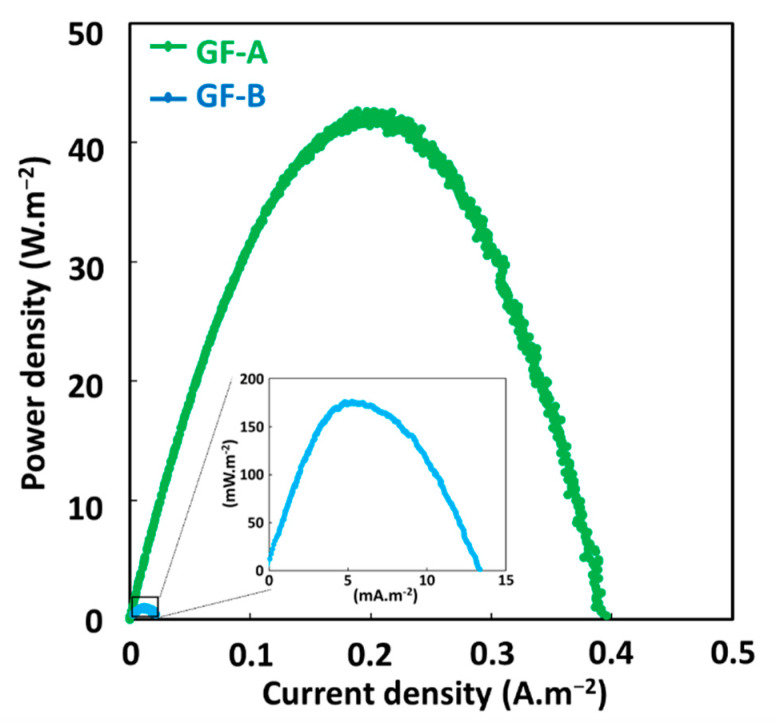
Power curves of the GF process obtained under GF-A (green line) and GF-B (blue line) conditions.

Figure 4 shows the MG degradation in the three comparative systems. As compared to the classical Fenton technique, the GF process not only leads to remarkable improvements in the initial degradation rate of MG in both conditions GF-A and GF-B, but also increases its maximum oxidation capacity (Table S2). In the CF process, a rapid initial MG decay is observed in the first minute, followed by a much slower degradation stage, which is due to the depletion of Fe^2+^ catalyst in the solution. The increase of the initial concentration of Fe^2+^ ions, increases both the initial degradation rate and the maximum oxidation capacity of CF process. Similar decay curves are obtained with GF-A process but with faster initial degradation rates and higher maximum degradation capacities for equivalent concentrations of total dissolved iron ions. The differences in degradation performances between CF and GF-A processes may be due to the formation of more reactive or more stable iron dissolved species in the case of ZVI-based Fenton, as previously reported [29,30]. The GF-B condition shows a faster and more complete MG degradation versus the GF-A condition for 1 min of immersion time. It indicated that the presence of anode and cathode electrodes during the treatment accelerate MG degradation. This result could be explained by the galvanic corrosion of ZVI electrode during the treatment that could enhance the release of total dissolved iron species. The presence of electrodes could also regenerate Fe^2+^ catalyst by reducing Fe^3+^ ions on copper cathode or on iron surface as previously reported [31,32]. For longer immersion times, the GF-A and GF-B conditions show similar degradation performances. By comparing both conditions, the longer the immersion time, the faster degradation of MG and the less the kinetics dependent on the presence of the electrodes. Power curves plotted in both conditions (Figure 5) show an important decrease in the maximal power density of GF process from 40 W·m^−2^ during the treatment (GF-B) to 175 mW·m^−2^ before the degradation process (GF-A). This result indicates that the Fenton reaction negatively affects the electrical production process, which involves a series of irreversible phenomena, such as activation overpotential due to electrochemical kinetics on the electrode surfaces, ohmic overpotentials associated with ohmic losses in both the ionic and electronic conductor, and concentration overpotentials due to the mass transfer limitations. Further study of the GF process based on individual anodic and cathodic compartments connected by a salt bridge will help us to understand the impact of the Fenton reaction on electrical energy production. This study shows that the GF-A configuration allows to produce more electrical energy but extends the duration of the treatment owing to long immersion times required to reach a sufficient concentration of Fe^2+^. The GF-B configuration allows for a reduction in the treatment duration but produces less electrical energy. Both configurations offer better degradation performances than classical Fenton and conventional ZVI-based Fenton. Moreover, electrical energy performances of the GF process obtained with both the GF-A and GF-B configuration are higher than electrical performances of microbial fuel cell technology, another electrochemical technology using bacteria to produce electrical energy from biodegradable organic molecules in wastewater with maximum power comprised between 1 mW·m^−2^ and 1 W·m^−2^ [33,34]. The electrical energy generated by the GF process can be harvested and exploited using an external circuit based on electronic power converters and digital processing devices that extract the maximum amount of electrical power and increase the voltage as needed.

### 2.3. Effect of Anode/Cathode Area Ratio on GF Process Performances

**Figure 6:**
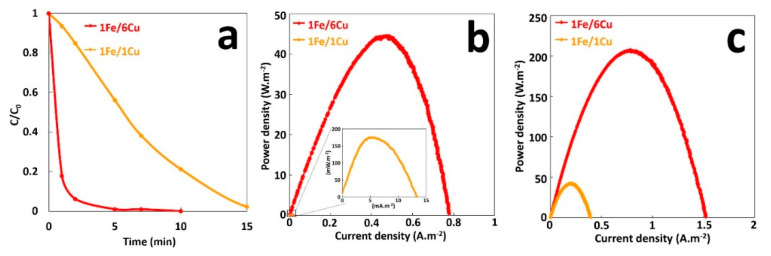
(**a**) Effect of Cu/Fe area ratio on the degradation of MG dye during the GF process, (**b**) Power curves of the GF process obtained with different Cu/Fe area ratios during degradation process (GF-B configuration), (**c**) Power curves of Fe/Cu couple without the Fenton reaction for different area ratios (GF-A configuration).

Another important factor in electrical energy obtained with galvanic cell is the effect of the surface area ratio of anode and cathode that directly affects galvanic current [23]. The Evans diagram indicates that the copper cathode is the rate-limiting electrode during galvanic corrosion. Thus, the larger the cathode compared with the anode, the more proton reduction can occur and the greater the galvanic current. To study the effect of the surface area ratio on GF process, the surface of copper area was changed from 6 to 36 cm^2^, with a constant anode surface area of 6 cm^2^. Degradation performances of the GF process for these copper/iron surface ratios, were studied in batch reactors, using 300-mL solutions of 10 mg·L^−1^ of MG at an initial pH of 3.00 in the presence of 2 mM H_2_O_2_ (Figure S3). As shown in Figure 6a, increasing the copper/iron surface ratio improves considerably the initial rate of MG degradation. Thus, initial degradation rate increases from 1.7 to 6 µM·min^−1^ by changing the surface ratio from 1 to 6, reducing the duration of 100% MG decolorization from 15 to 5 min (Table S3). This is very likely to result from the enhancement of the galvanic corrosion rate, and hence, the concentration increases of total dissolved iron released in solution. Power curves plotted during the treatment in the presence of H_2_O_2_ (Figure 6b) show an important increase in the maximal power density of GF process from 175 mW·m^−2^ to 45 W·m^−2^ by increasing the cathode surface from 6 to 36 cm^2^. This sharp increase in power cannot be only due to the rise of corrosion current density. Moreover, as shown in Figure 6c, electrical energy production of electrochemical cells in the absence of Fenton reaction (without H_2_O_2_) is higher. Thus, 40 and 200 W·m^−2^ of P_max_ are obtained with 6 and 36 cm^2^ of cathode areas, respectively. In this last case, the increase of P_max_ is only due to the rise of corrosion current density since it is directly proportional to the cathode surface. In what follows, the effect of operating parameters on the performance of GF process will be analyzed with GF-A configuration, since it allows to produce more electrical energy.

### 2.4. Effect of pH on GF Process Performances

**Figure 7:**
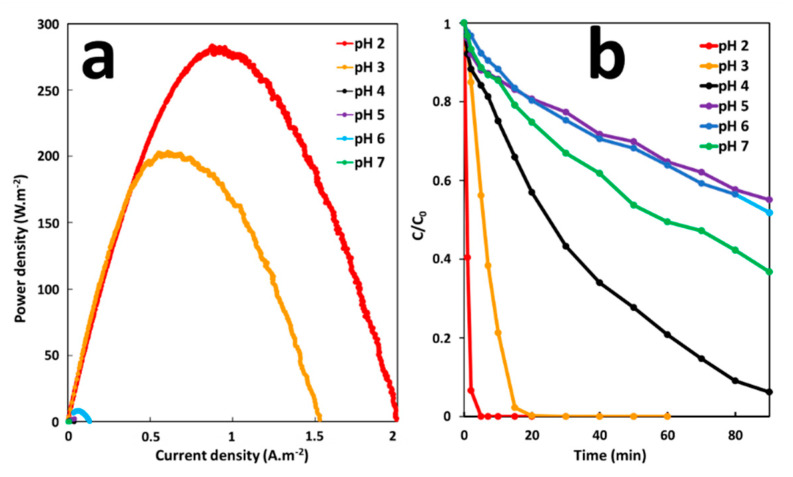
(**a**) Power curves of the GF process obtained at different pH and (**b**) Effect of pH on the degradation of MG dye during the GF process.

The effect of pH on the performance of the GF process was studied under the GF-A configuration at a pH range from 2 to 7. The pH of the solution was adjusted to the desired level by using sulfuric acid. The effect of pH on energy production was studied before degradation in batch reactors, by galvanic corrosion of an iron plate (6 cm^2^ of immersed surface) electrically connected to a copper cathode (36 cm^2^ of immersed surface) in 300-mL solutions of 10 mg·L^−1^ MG. Degradation performances were studied by removing iron anode and copper cathode from solutions after 5 min of immersion time and by adding H_2_O_2_ at a final concentration of 2 mM to initiate the degradation reaction. As shown in Figure 7a,b, the maximum power density (200–280 mW·m^−2^) and the maximum initial degradation rate of MG (20–77 µM·min^−1^) are obtained at a lower acidic pH range (2–3). Increasing the pH range, from 4 to 7, considerably decreases the maximal power density (0.1–10 mW·m^−2^) and the initial degradation rate (0.4–2.3 µW·m^−2^). This sharp decrease in power and degradation efficiency, is probably due to the transformation of ferric and ferrous ions into insoluble amorphous hydroxides at higher pH. Precipitation of amorphous hydroxides can lead to electrode passivation. The kinetic pathways of iron electrode transformations in the GF process and the mechanistic of in situ iron catalyst formation were described in a previous work [21]. In addition, the increase in pH slows down the proton reduction kinetics at the cathode. Indeed, hydrogen reduction rates are faster in acidic environments [35]. At higher pH, the corrosion current density could decrease since it is directly proportional to the hydrogen reduction rate at the cathode surface. Like the Fenton oxidation, the optimum initial pH of MG degradation for the GF process ranges between 2 and 3. At higher pH, hydrogen peroxide is unstable and may decompose losing its oxidation ability [36]. Overall, it can be concluded that the maximum efficiency of the GF process toward MG degradation can be achieved at pH 2–3.

### 2.5. Effect of Temperature on GF Process Performances

**Figure 8:**
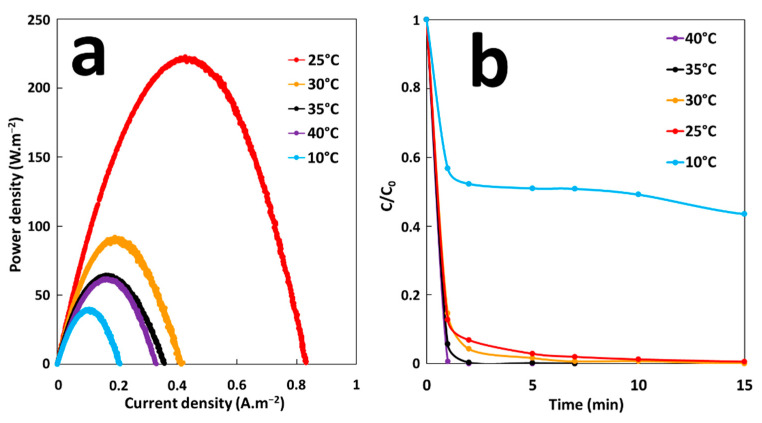
(**a**) Power curves of the GF process obtained at different temperatures and (**b**) Effect of temperature on the degradation of MG dye during the GF process.

The effect of temperature on energy production and MG degradation rate of GF process was studied under the GF-A configuration at a temperature range from 10 to 40 °C. The temperature of MG solutions (300-mL solutions of 10 mg·L^−1^ MG) was adjusted in batch reactors. Energy production was studied by immersion of an iron plate (6 cm^2^ of immersed surface) electrically connected to a copper cathode (36 cm^2^ of immersed surface) in solutions. Degradation performances were studied by removing electrodes from solutions after 5 min of immersion time and by adding H_2_O_2_ at a final concentration of 2 mM. According to the Figure 8a, maximum energy production is obtained at 25 °C (P_max_ = 220 mW·m^−2^). The maximum power density, P_max_, decreases to 40 mW·m^−2^ by decreasing the temperature to 10 °C. While the rise in temperature from 25 to 30 °C decreases P_max_ to 90 mW·m^−2^. From 35 °C, P_max_ reaches a threshold value of 65 mW·m^−2^. A general explanation of observed temperature effects on energy production cannot be made because of the variety of influences that temperature has on several components of the galvanic system. Energy production depends on the redox reaction rates, galvanic potential, and conductivity of both ionic electrolyte solution and metallic electrodes. Each of these parameters responds to temperature change. With rise in temperature the overvoltage for hydrogen reduction on cathode diminishes and the rate of galvanic corrosion increases [37]. Ionic conductivity of the electrolyte solution also increases with increase of temperature [38]. One or both parameters are probably predominant in the energy production process at low temperature, leading to the increase of P_max_ with the rise in temperature from 10 to 25 °C. The electrical conductivity of metallic electrodes decreases with increase of temperature [39]. Increasing temperature also accelerates self-corrosion rate of electrodes [40]. The galvanic potentials change with temperature and reversal of polarity is possible, if the potentials of the coupled metals change unevenly enough [41]. The way in which each of these units responds to temperature change, could explain the decrease in energy production for temperatures above 25 °C. Figure 8b shows the extent of MG degradation as function of time at different temperatures. It is clear that the rate of MG degradation increases by increasing temperature. At 10 °C, the degradation rate is much lower compared with the values at higher temperatures. After 15 min of degradation time, MG decolorization at 10 °C was about 57%, while 100% decolorization was achieved at higher temperatures. These results indicate that initial concentration of Fe^2+^ ions at 10 °C was much lower compared with higher temperatures because of decrease in galvanic corrosion rate. Based on these results, it can be concluded that the maximum efficiency of the GF process can be obtained at 25 °C.

### 2.6. Effect of H_2_O_2_ Concentration on GF Process Performances

**Figure 9:**
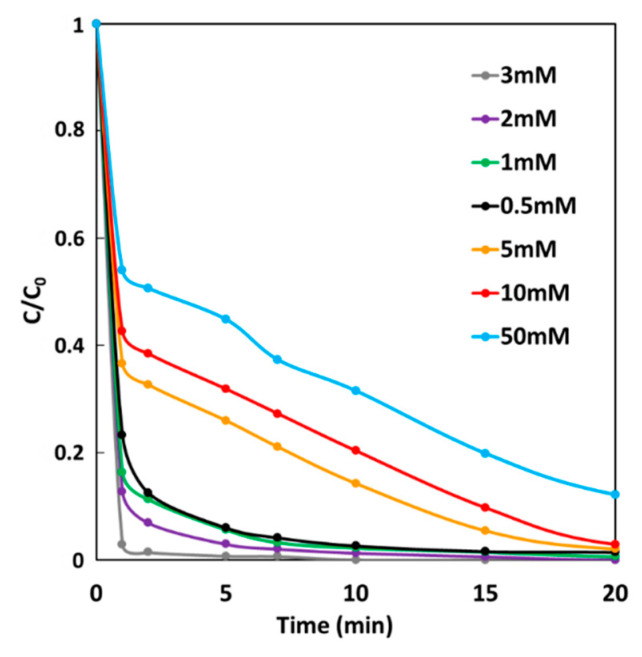
Effect of H_2_O_2_ concentration on the degradation of MG dye during the GF process.

The effect of H_2_O_2_ concentration on MG degradation rate of the GF process was studied under GF-A condition at 25 °C. In a batch reactor, an iron plate (6 cm^2^) electrically connected to a copper cathode (36 cm^2^) were immersed in 300-mL solutions of 10 mg·L^−1^ MG. Degradation performances were studied by removing electrodes from solutions after 5 min of immersion time and by adding H_2_O_2_ at a final concentration range from 0.5 to 50 mM. Figure 9 shows MG degradation rates as a function of time at different initial H_2_O_2_ concentrations, which increases with increasing H_2_O_2_ concentration from 0.5 to 3 mM. In this concentration range, 98% decolorization is achieved after less than 10 min. For higher H_2_O_2_ concentrations, MG degradation rate decreases leading to 99%, 98%, and 88% decolorization for 5, 10 and 50 mM, respectively, after 1 h. An optimal H_2_O_2_ concentration has already been reported in the Fenton oxidation of dyes [42,43]. Indeed, for high concentrations, H_2_O_2_ excess acts as a scavenger of hydroxyl radical (^•^OH), resulting in less dye degradation. For these conditions of copper/iron surface ratio and iron surface/solution volume ratio, the maximum efficiency of GF process can be obtained at the optimal H_2_O_2_ concentration of 3 mM.

### 2.7. Effect of Initial MG Concentration on GF Process Performances

**Figure 10:**
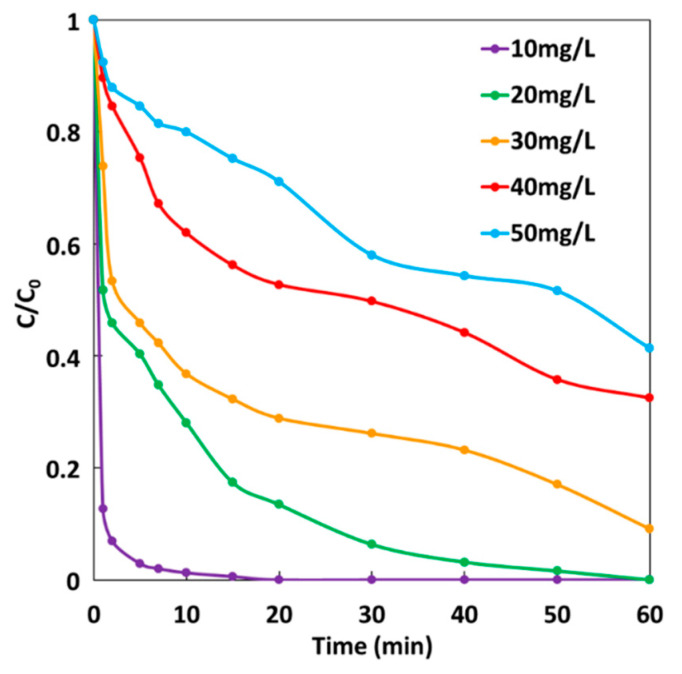
Effect of MG concentration on the degradation rate during the GF process.

The effect of initial dye concentration on the degradation efficiency of MG dye was studied under GF-A configuration according to the same experiment described to study the effect of H_2_O_2_ concentration. H_2_O_2_ was added at a final concentration of 2 mM. Figure 10 shows MG degradation rates at five different initial MG concentrations ranging from 10 to 50 mg·L^−1^. It can be seen that the rate of MG degradation decreases when the initial concentration of the dye increases, which is mainly due to a higher number of dye molecules available for reaction. Thus, degradation efficiency decreases considerably with an increase in the MG concentration. Indeed, 100% decolorization of 10 mg·L^−1^ is achieved after 20 min, while 100% decolorization of 20 mg·L^−1^ is obtained after 1 h. For higher initial concentrations of MG, total decolorization of solutions is never achieved after 1 h, leading to 91%, 68%, and 59% decolorization of 30, 40, and 50 mg·L^−1^ concentrations of MG, respectively. It can be concluded that under these experimental conditions, the GF process can achieve total decolorization of MG solutions for concentrations below 20 mg·L^−1^ in less than 1 h.

## 3. Materials and Methods

### 3.1. Materials

Reagent grade hydrogen peroxide (30 wt.% solution), iron(II) sulfate heptahydrate (FeSO_4_·7H_2_O), sulfuric acid, sodium hydroxide, and malachite green were all purchased from Sigma-Aldrich (Vienne, France). Iron, stainless steel, copper, and graphite plates were purchased from GoodFellow (Lille, France).

### 3.2. Setup and Operation

To study the effect of cathode materials and to compare the performance of GF to classical Fenton process, batch process experiments were carried out in a Pyrex glass reactor (Labbox, Rungis, France) of 250 mL containing 100 mL of malachite green (10 mg·L^−1^). Solutions were adjusted to pH 3 using sulfuric acid and iron(II) sulfate heptahydrate was added for the classical Fenton study. Prior to each GF process experiment, electrodes were mechanically polished with SiC papers up to 4000 grade and then cleaned with distilled water and dried with warm air. All experiments were initiated by addition of a known amount of hydrogen peroxide. The solution in the reactor was thoroughly stirred (200 rpm) with a magnetic stirrer to ensure complete mixing. The reaction temperature was kept constant at the room temperature (25 °C). A Pyrex glass reactor of 500 mL was used to study the effect of cathode/anode area ratios.

### 3.3. Monitoring MG Degradation

The efficiency of MG degradation was evaluated by measuring absorbance at 619 nm using a spectrophotometer (DR 3900, Hach-Lange, Marne La Vallée, France). The concentration of the MG in the reaction mixture at different reaction times was determined by measuring the absorption intensity at λ_max_ = 619 nm and from a calibration curve. Prior to the measurement, a calibration curve was obtained by using the standard MG solution with known concentrations. The decolorization percentage was calculated using the following equation:

(3) $$\%\;Decolorization=\frac{C_{0}-C_{t}}{C_{0}}\times 100$$

where *C*_0_ is the initial concentration of the dye (µM) and *C_t_* is its concentration (µM) at any time *t* (min). Spectrophotometry was applied to characterize the MG decolorization kinetics. The kinetics study was carried out using Chu’s model expressed by Equation (4) [44].

(4) $$\frac{C_{t}}{C_{0}}=1-\frac{t}{\left[p+a\times t\right]}$$

where *a* and *p* are two constants related to initial reaction rate and maximum oxidation capacity, respectively. 1/*p* depends on the initial decolorization rate (−*r*_0_) according to Equation (5).

(5) $$\left(-r_{0}\right)=C_{0}\times (1/p)$$

where 1/*a* represents the maximum oxidation capacity beyond which no higher degradation can be achieved. The Chu’s kinetics model suitably describes the decolorization kinetics of dyes by Fenton reaction in both homogeneous and heterogeneous systems [45]. More details on operating parameters and model kinetic parameters are described in supporting information section.

### 3.4. Determination of Total Dissolved Iron Ions

The concentrations of total dissolved iron ions (the sum of ferrous ions and ferric ions) were analyzed using the FerroVer© test purchased from Hach-Lange (Marne La Vallée, France). This analysis was based on 1,10-phenanthroline method. The reagent in this test procedure converts all soluble iron and most insoluble forms of iron in the sample to soluble ferrous iron for measurement. The ferrous iron reacts with the 1–10 phenanthroline indicator in the reagent to form an orange color (510 nm) in proportion to the iron concentration. DR 3900 spectrophotometer (Hach, Marne La Vallée, France) was used to measure the wavelength and to quantify total dissolved iron ions produced by galvanic corrosion of an iron plate (6 cm^2^ of immersed surface) electrically connected to a copper cathode (6 cm^2^ of immersed surface) submerged in aqueous solutions (100 mL) at an initial pH of 3.00. Figure S4 shows the concentration of total dissolved iron ions determined for different immersion times.

### 3.5. Corrosion Characterization

The galvanic corrosion of metal couples was investigated by Evans diagrams plotted using linear sweep voltammetry (LSV) utilizing a three-electrode arrangement at a scanning rate of 10 mV·s^−1^ in aqueous solutions acidified with sulfuric acid at pH of 3.00. A potentiostat OGS 500 from Origalys was used to perform electrochemical characterizations. The metal electrodes (6 cm^2^) were used as working electrodes, a commercial saturated Ag/AgCl electrode as a reference and a Pt wire electrode as an auxiliary electrode. The geometric surface area of metal electrodes was used for calculating galvanic corrosion current density. These diagrams allow for identification of E_corr_ and i_corr_, where the anodic and cathodic reactions proceed with the same rate.

### 3.6. Polarization Curve Measurement

The polarization curves of the GF processes were measured by LSV utilizing a two-electrode arrangement with a potentiostat (OGS 500 from Origalys, Rillieux-la-Pape, France). LSV was performed from the open circuit potential to 0 V using a scan rate of 10 mV·s^−1^. The power was calculated by multiplying the current by the voltage. The geometric surface area of iron electrode was used for calculating power density of GF process.

## 4. Conclusions

In summary, a new approach to produce electrical energy in the Galvano–Fenton process was successfully developed. Simultaneous MG degradation and electrical energy production were investigated in a batch process. The investigation of various design parameters emphasizes that utilization of copper as a low-cost cathode material that provides good treatment and electrical production performances. Moreover, these performances are improved by increasing the surface area of the cathode (220 mW·m^−2^). Degradation efficiency was also investigated with the influence of process parameters including pH, temperature, and the concentrations of H_2_O_2_ and MG dye. The optimal conditions included a pH range of 2–3, a temperature of 25 °C, and an H_2_O_2_ concentration of 3 mM. The results indicated that GF process was operative as a color degradation efficiency of 100% attained within 30 min with initial MG concentration of 10 mg/L, and 98% with initial MG concentration of 20 mg/L obtained after 1 h. These interesting performances indicate that this new Energy-from-Waste approach of the GF process has promising application potential as an advanced oxidation process for wastewater treatment, but also for scrap iron recovering and production of electrical energy as well as chemical energy via cathodic formation of hydrogen.

## Data Availability

The data presented in this study are available in Supplementary Materials.

## Supplementary Materials

The following are available online. Figure S1: schematic illustration of the experience studying the performance of the GF process with different cathode materials; Figure S2: schematic illustration of the experience studying the performance of the GF process under two conditions in comparison with classical Fenton process; Figure S3: schematic illustration and photos of the experience studying the effect of anode/cathode area ratio on GF process performances; Figure S4: Total dissolved iron ions determined for different immersion times; Table S1: Chu’s model parameters of MG degradation kinetics with GF process using different cathode materials; Table S2: Chu’s model parameters of MG degradation kinetics with CF, GF-A and GF-B processes; Table S3: Chu’s model parameters of MG degradation kinetics with GF process using different cathode/anode area ratios; Table S4: Chu’s model parameters of MG degradation kinetics with GF process in different pH.

## Sample Availability

Samples of the compounds are not available from the authors.
