# Supplementary material for: A Novel Energy-from-Waste Approach for Electrical Energy Production by Galvano–Fenton Process
Source: Molecules. 2021 Jun 30;26(13):4013. doi: 10.3390/molecules26134013 (PMC8271935; doi:10.3390/molecules26134013)
Supplement: Supplementary file 1 [file molecules-26-04013-s001.zip › molecules-1271492-SI.pdf]

Supporting Information

A novel Energy-from-Waste approach for production of electrical energy by Galvano-Fenton process

Intissar Gasmi [a, b], Naoufel Haddour [a*], Oualid Hamdaoui [b, c], Kaouther Kerboua [b, d], Abdulaziz Alghyamah [c], François Buret [a]

[a] Laboratoire Ampère, École Centrale de Lyon, 36 Avenue Guy de Collongue, 69134 Écully, France

[b] Laboratory of Environmental Engineering, Process Engineering Department, Faculty of Engineering, Badji Mokhtar - Annaba University, P.O. Box 12, 23000 Annaba, Algeria

[c] Chemical Engineering Department, College of Engineering, King Saud University, P.O. Box 800, 11421 Riyadh, Saudi Arabia

[d] Higher School of Industrial Technologies, Department of Second Cycle, P.O. Box 218, 23000 Annaba, Algeria

* Corresponding author. Email: naoufel.haddour@ec-lyon.fr

The Chu's kinetics model.

The Chu's kinetics model is characterized by two constants, a and p, and is written as follows:

$$\frac{C_t}{C_0} = 1 - \frac{t}{[p + a \times t]} \quad (2)$$

where C_0 (μM) is the initial concentration of the dye and C_t (μM) is its concentration at any time t (min). Eq. 2 can be rearranged to a linearized form:

$$\frac{t}{\left[1 - \frac{C_t}{C_0}\right]} = p + a \times t \quad (3)$$

A plot of $\frac{t}{\left[1-\frac{C_t}{C_0}\right]}$ versus t results in a straight line with an intercept p and a slope a.

p and a are two constants concerning initial reaction rate and maximum oxidation capacity, respectively. 1/p is related to its initial decolorization rate ($-r_0$) expressed as eq.4.

$$(-r_0) = C_0 \times (1/p) \quad (4)$$

1/a represents the maximum oxidation capacity beyond which no higher degradation can be achieved. The Chu's kinetics model suitably describes the decolorization kinetics of dyes by Fenton reaction in both homogeneous and heterogeneous systems.

Kinetics model parameters.

Table S1. Chu's model parameters of MG degradation kinetics with GF process using different cathode materials.

System	Chu parameters			
	1/p (min^{-1})	1/a	R^2	r_0 ($\mu mol\ L^{-1}\ min^{-1}$)
Fe	0.21	1.36	0.98	5.83
Fe-Cu	0.70	1.15	0.99	19.32
Fe-SS	0.41	1.17	0.99	11.38
Fe-C	0.21	1.22	0.97	5.98

Table S2. Chu's model parameters of MG degradation kinetics with CF, GF-A and GF-B processes.

Condition	System	Chu parameters			
		1/p (min^{-1})	1/a	R^2	r_0 ($\mu mol\ L^{-1}\ min^{-1}$)
2.69 mg L^{-1} Eq. 1 min	CF	0.06	0.13	0.96	1.73
	GF-A	0.13	0.39	0.95	3.77
	GF-B	0.52	0.81	0.99	14.38
8 mg L^{-1} Eq. 5 min	CF	0.24	0.58	0.96	6.77
	GF-A	0.50	0.81	0.98	13.91
	GF-B	0.69	0.78	0.98	18.99
11.8 mg L^{-1} Eq. 10 min	CF	0.60	0.76	0.99	16.57
	GF-A	14.49	1.00	1	397.09
	GF-B	16.97	1.00	1	465.19
17.8 mg L^{-1} Eq. 20 min	CF	2.10	1.01	0.99	57.68
	GF-A	98.03	1.00	1	2686.27
	GF-B	42.55	1.00	1	1165.95

Table S3. Chu's model parameters of MG degradation kinetics with GF process using different cathode/anode area ratios.

Cathode/Anode area ratio	Chu parameters			
	1/p (min^{-1})	1/a	R^2	r_0 ($\mu mol\ L^{-1}\ min^{-1}$)
1	0.06	0.47	0.99	1.76
6	0.21	1.02	0.99	5.98

.

Table S4. Chu's model parameters of MG degradation kinetics with GF process in different pH.

pH	Chu parameters			
	1/p (min^{-1})	1/a	R^2	r_0 (μmol L^{-1} min^{-1})
2	2.82	1.03	0.99	77.37
3	0.70	1.15	0.99	19.32
4	0.085	0.30	0.92	2.32
5	0.051	0.32	0.95	1.39
6	0.016	0.40	0.99	0.44
7	0.043	0.22	0.97	1.17

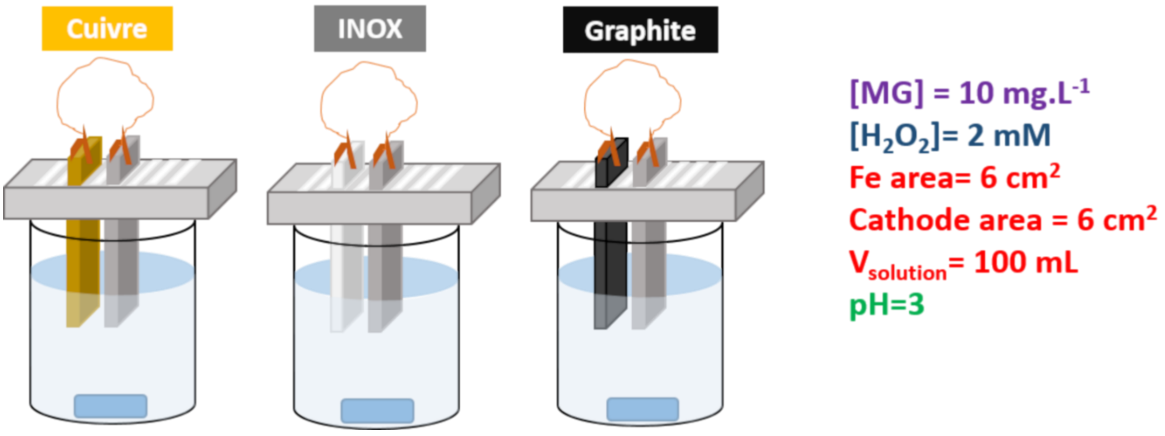

Figure S1. schematic illustration of the experience studying the performance of the GF process with different cathode materials.

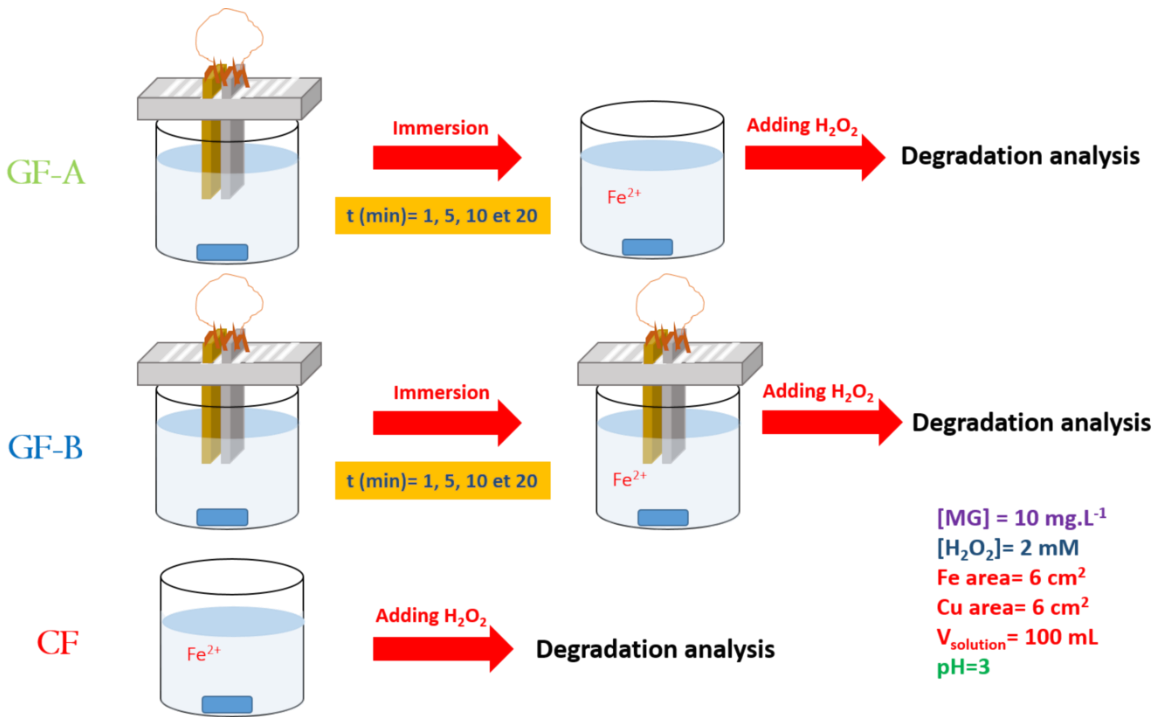

Figure S2 schematic illustration of the experience studying the performance of the GF process under two conditions in comparison with classical Fenton process.

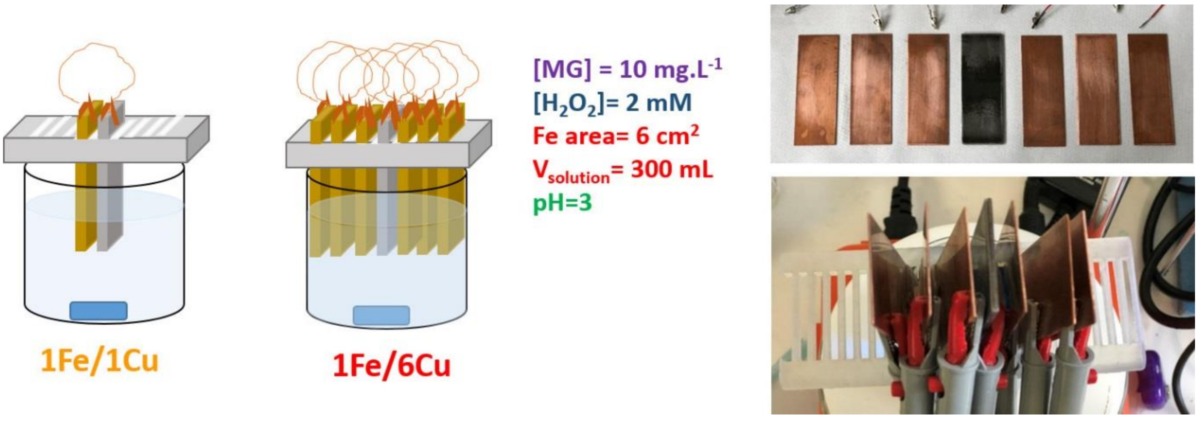

Figure S3 schematic illustration and photos of the experience studying the effect of anode/cathode area ratio on GF process performances.

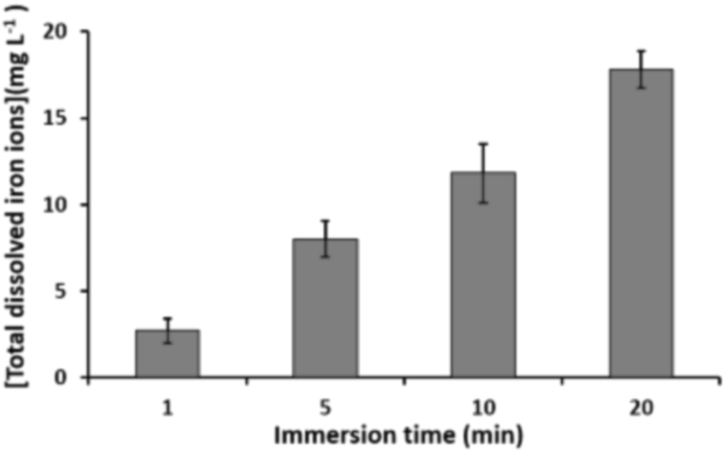

Figure S4. *Total dissolved iron ions determined for different immersion times.*
